# Eclampsia

**DOI:** 10.21980/J8M93D

**Published:** 2020-07-15

**Authors:** Patrick G Meloy, Megan C Henn, Daniel Rutz, Amit Bhambri

**Affiliations:** * Emory University School of Medicine, Department of Emergency Medicine, Atlanta, GA; ^ University of Wisconsin-Madison School of Medicine and Public Health, Department of Emergency Medicine, Madison, WI; † Swedish Covenant Hospital, Department of Emergency Medicine, Chicago, IL

## Abstract

**Audience:**

Emergency medicine residents and medical students on emergency medicine rotations.

**Introduction:**

Eclampsia is an uncommon but important life-threatening obstetrical emergency, complicating 1.5–10 deliveries per 10,000 pregnancies in resource-rich countries.^1^ If not recognized and treated promptly, there is risk of significant morbidity or death to both mother and baby. Clinically, eclampsia is defined by new-onset seizures or coma in women with preeclampsia.^2^ Preeclampsia is defined by maternal hypertension after 20 weeks gestation with or without signs of end organ dysfunction, and, like eclampsia, can develop in the postpartum period.^1^ Eclampsia manifests as new onset generalized tonicclonic seizures. Eclamptic seizures are usually preceded by neurologic symptoms such as severe or atypical headache, visual disturbances, and non-neurologic symptoms such as severe abdominal pain or proteinuria.^1^ Emergent treatment involves prompt administration of (intravenous) IV magnesium sulfate.^2^,^3^,^4^ Adjuncts include securing the airway if necessary and administration of IV antihypertensive medications. Like preeclampsia, definitive management is by prompt delivery of the fetus if the mother is still pregnant.^1^ If untreated, maternal mortality is as high as 14%.^1^ Women who develop eclampsia are at increased risk of obstetric complications in subsequent pregnancies and at higher risk for cardiovascular disease and metabolic disease later in life.

**Educational Objectives:**

At the end of this oral boards session, examinees will: 1) Demonstrate ability to obtain a complete medical history including a detailed obstetric history. 2) Demonstrate the ability to perform a detailed physical examination in a postpartum female patient who presents with a seizure. 3) Investigate the broad differential diagnoses which include electrolyte imbalances, brain tumor, meningitis or encephalitis, hemolysis, elevated liver enzymes, low platelets (HELLP) syndrome and eclampsia. 4) List the appropriate laboratory and imaging studies to differentiate eclampsia from other diagnoses (complete blood count, comprehensive metabolic panel, magnesium level, pregnancy testing, urinalysis, and computed tomography [CT] scan of the head). 5) Identify a postpartum eclampsia patient and manage appropriately (administer IV magnesium therapy, administer IV antihypertensive therapy, emergent consultation with an obstetrician). 6) Provide appropriate disposition to the intensive care unit after consulting with an obstetrician.

**Educational Methods:**

This was envisioned as an oral board testing case due to the multiple aspects which require emergency care. Residents are expected to assess the seriousness of the patient’s condition, elicit critical details from her recent medical history, and synthesize that data in order to treat a medically complex patient. Oral board testing is able to incorporate each of these aspects together and provide the resident with a dynamic learning environment.

Oral board testing is a way to assess the resident’s ability to rapidly obtain and interpret multiple sources of information simultaneously. By utilizing a case that requires pharmaceutical therapy, the clinical competency committee is able to obtain additional milestones which are sometimes difficult to test in the emergency department itself.

Learners were assessed using online evaluation tools available, ie, Google forms. Critical actions were subsequently tied to Emergency Medicine Milestones and the results were compiled and used for resident evaluations and clinical competency. Residents were given verbal feedback immediately after the examination, and they were provided with the scores of their online evaluation after all results were compiled.

**Research Methods:**

Learners and instructors provided written feedback after the case was administered to assess for strengths and weaknesses of the case, and modifications were then made to better address concerns. Learners answered written multiple-choice questions on high-level concepts, ie, critical actions, at least one month after this exam was completed.

**Results:**

Learners found this a challenging, but enjoyable, way to refresh their knowledge and skills regarding preeclampsia, and this was a highly rated part of their mock oral board examination. Overall, residents rated the session 4.3 (1–5 Likert scale, 5 being Excellent) after the oral board review session was completed. Comments from residents included “haven’t seen post-partum preeclampsia in residency” and “challenging to remember magnesium dosing.”

**Discussion:**

Residents and medical students were evaluated using this method and both enjoyed the activity as a novel way to study as well as exercise their medical knowledge. The content was both highly relevant to the practice of emergency medicine and the format was an effective way to deliver the information to the learners. The case is a good model to evaluate for the high stakes testing of both the written and oral board examinations, but also a way to assess residents’ abilities to treat preeclamptic and eclamptic patients in the emergency department.

**Topics:**

Eclampsia, preeclampsia, seizures, end-organ damage, hypertensive emergency, altered mental status, neurologic emergency, obstetric emergency.

## USER GUIDE

[Table t1-jetem-5-3-o1]

**Table t1-jetem-5-3-o1:** 

List of Resources:	
Abstract	1
User Guide	4
For Examiner Only	6
Oral Boards Assessment	12
Stimulus	15
Debriefing and Evaluation Pearls	27

**Learner Audience:**

Medical students, interns, junior residents, senior residents

**Time Required for Implementation:**

Case: 15 minutes as a single case, 10 minutes if used as triple case

Debriefing: 10 minutes

**Learners per instructor:**

Recommend 1 learners per instructor/case, if using as oral board testing

**Topics:**

Eclampsia, preeclampsia, seizures, end-organ damage, hypertensive emergency, altered mental status, neurologic emergency, obstetric emergency.

**Objectives:**

By the end of this oral boards session, examinees will:

1. Demonstrate ability to obtain a complete medical history including a detailed obstetric history.
2. Demonstrate the ability to perform a detailed physical examination in a postpartum female patient who presents with a seizure.
3. Investigate the broad differential diagnoses which include electrolyte imbalances, brain tumor, meningitis or encephalitis, HELLP syndrome and eclampsia.
4. List the appropriate laboratory and imaging studies to differentiate eclampsia from other diagnoses (complete blood count, basic metabolic panel, magnesium level, pregnancy testing, urinalysis, and CT scan of the head).
5. Identify a postpartum eclampsia patient and manage appropriately (administer IV magnesium therapy, administer IV antihypertensive therapy, emergent consultation with an obstetrician).
6. Provide appropriate disposition to intensive care unit (ICU) after consulting with an obstetrician.

### Linked objectives and methods

The learner in this case must be able to synthesize available historical and physical examination (Objectives 1 and 2) data in order to develop a broad list of differential diagnoses for a postpartum patient presenting with a seizure (Objective 3). Without performing a thorough history and physical examination, the final diagnosis may be missed if the learner does not identify that the patient is postpartum with clinical signs of eclampsia (Objective 3, 4 and 5). The oral board formatting allows the learner to synthesize real-time data in order to differentiate eclampsia from electrolyte disorders, infectious etiologies or cerebral space-occupying lesions. The learner must be able to identify eclampsia and provide timely and appropriate treatment and disposition to prevent morbidity (Objectives 5 and 6). Debriefing of the case immediately afterward ensures assimilation of the sources of data in order to obtain the correct diagnosis and appropriate management of the case.

### Recommended pre-reading for instructor

- Cassella C, Koyman A, Long, B. Elemental EM: preeclampsia. emDocs. http://www.emdocs.net/elemental-em-preeclampsia/. Published December 2017. Accessed March 10, 2020.
- Townsend R, O’Brien P, Khalil A. Current best practice in the management of hypertensive disorders in pregnancy. *Integr Blood Press Control*. 2016; 9:79–94. doi:10.2147/IBPC.S77344.
- LaFollette, R. Preeclampsia in the ED. Taming the SRU. http://www.tamingthesru.com/blog/diagnostics/preeclampsia. Published November 2018. Accessed March 10, 2020.

### Results and tips for successful implementation

This model is best implemented as an oral board examination. The learner should be directly observed by the examiner, with the option of having additional learners or instructors observing the case progression. This was tested during mock oral board simulation, as well as during oral board practice sessions. Assessment forms were created online using Google documents (http://docs.google.com/forms). The forms measured critical actions, which were then tied to Emergency Medicine Milestones on the backend of the questions (https://www.abem.org/public/docs/default-source/defaultdocument-library/em-milestones.pdf?sfvrsn=e627c8f4_0). In this way, the oral board formatting could be used to both assess a resident’s clinical knowledge of an emergent condition, but also to evaluate their progress along the emergency medicine milestones.

Initially, learners were not provided with information about the patient being postpartum, but this caused the majority of the junior learners to miss the diagnosis, and so the patient’s husband became available to provide this information. We deliberately did not include serum or urine pregnancy testing in this scenario because it is likely to be negative at three-weeks and is a highly confounding variable. Overall, this case is a good way to assess the learner’s ability to think quickly, make efficient medical decisions, and perform under pressure. This case was well-received by learners and was felt to be a good assessment of their evaluation skills. Pregnant patients are infrequently evaluated in this institution’s emergency department, and learners found this type of simulated exam a good way to evaluate their knowledge and skill set.

After the overall examination was completed (two single cases and a triple case were administered), learners rated the oral boards session using a Likert scale (1–5, 5 being Excellent), and this case received an overall 4.3 by 37 learners. Comments such as “haven’t seen post-partum preeclampsia in residency,” “challenging to remember magnesium dosing,” and “easy diagnosis, but I forgot to test deep tendon reflexes” were provided. Finally, we feel that this is a highly testable concept on the emergency medicine written and oral board certification exams.

## Figures and Tables

**Table t2-jetem-5-3-o1:** 

**Vitals:** HR 107	BP 177/103	RR 22	Temp 37.1°C	O_2_Sat 98%

**Table t3-jetem-5-3-o1:** 

	Milestone	Did not achieve level 1	Level 1	Level 2	Level 3
**1**	**Emergency Stabilization (PC1)**	□ **Did not achieve Level 1**	□ **Recognizes abnormal vital signs**	□ **Recognizes an unstable patient, requiring intervention** **Performs primary assessment** **Discerns data to formulate a diagnostic impression/plan**	□ **Manages and prioritizes critical actions in a critically ill patient** **Reassesses after implementing a stabilizing intervention**
**2**	**Performance of focused history and physical (PC2)**	□ **Did not achieve Level 1**	□ **Performs a reliable, comprehensive history and physical exam**	□ **Performs and communicates a focused history and physical exam based on chief complaint and urgent issues**	□ **Prioritizes essential components of history and physical exam given dynamic circumstances**
**3**	**Diagnostic studies (PC3)**	□ **Did not achieve Level 1**	□ **Determines the necessity of diagnostic studies**	□ **Orders appropriate diagnostic studies** **Performs appropriate bedside diagnostic studies/procedures**	□ **Prioritizes essential testing** **Interprets results of diagnostic studies** **Considers risks, benefits, contraindications, and alternatives to a diagnostic study or procedure**
**4**	**Diagnosis (PC4)**	□ **Did not achieve Level 1**	□ **Considers a list of potential diagnoses**	□ **Considers an appropriate list of potential diagnosis** **May or may not make correct diagnosis**	□ **Makes the appropriate diagnosis** **Considers other potential diagnoses, avoiding premature closure**
**5**	**Pharmacotherapy (PC5)**	□ **Did not achieve Level 1**	□ **Asks patient for drug allergies**	□ **Selects an appropriate medication for therapeutic intervention, considering potential adverse effects**	□ **Selects the most appropriate medication(s) and understands mechanism of action, effect, and potential side effects** **Considers and recognizes drug-drug interactions**
**6**	**Observation and reassessment (PC6)**	□ **Did not achieve Level 1**	□ **Reevaluates patient at least one time during the case**	□ **Reevaluates patient after most therapeutic interventions**	□ **Consistently evaluates the effectiveness of therapies at appropriate intervals**
**7**	**Disposition (PC7)**	□ **Did not achieve Level 1**	□ **Appropriately selects whether to admit or discharge the patient**	□ **Appropriately selects whether to admit or discharge** **Involves the expertise of some of the appropriate specialists**	□ **Educates the patient appropriately about their disposition** **Assigns patient to an appropriate level of care (ICU/Tele/Floor)** **Involves expertise of all appropriate specialists**
**22**	**Patient centered communication (ICS1)**	□ **Did not achieve level 1**	□ **Establishes rapport and demonstrates empathy to patient (and family)** **Listens effectively**	□ **Elicits patient’s reason for seeking health care**	□ **Manages patient expectations in a manner that minimizes potential for stress, conflict, and misunderstanding.**
**23**	**Team management (ICS2)**	□ **Did not achieve level 1**	□ **Recognizes other members of the patient care team during case (nurse, techs)**	□ **Communicates pertinent information to other healthcare colleagues**	□ **Communicates a clear, succinct, and appropriate handoff with specialists and other colleagues** **Communicates effectively with ancillary staff**

**Table t4-jetem-5-3-o1:** 

**#1**	**Patient information form**
**#2**	**Arterial blood gas**
**#3**	**Complete blood count (CBC)**
**#4**	**Basic metabolic panel (BMP)**
**#5**	**Urinalysis**
**#6**	**Chest radiograph**
**#7**	**Head CT**
**#8**	**Electrocardiogram (ECG)**
**#9**	**Toxicology (serum and urine)**
**#10**	**Coagulation panel**
**#11**	**Liver function panel**

**Table t5-jetem-5-3-o1:** 

**Patient’s Name:**	**Shannon White**
**Age:**	**29**
**Gender:**	**F**
**Chief Complaint:**	**Seizure**
**Person Providing History:**	**Husband**

**Table t6-jetem-5-3-o1:** 

**Vital Signs:**	
**Temp:**	**37.1°C**
**BP:**	**177/103**
**P:**	**107**
**RR:**	**22**
**O**_**2**_**sat:**	**98% (room-air)**
**Weight:**	**79 kg**

**Table t7-jetem-5-3-o1:** 

**pH**	**7.39**
**pCO2**	**41 mmHg**
**pO2**	**88 mmHg**
**HCO3**	**22 mmol/L**
**O2**	**sat 97%**

**Table t8-jetem-5-3-o1:** 

**White blood cell count (WBC)**	**12.2 ×1000/mm3**
**Hemoglobin (Hgb)**	**9.2 g/dL**
**Hematocrit (Hct)**	**28.8%**
**Platelets**	**350 ×1000/mm3**

**Table t9-jetem-5-3-o1:** 

**Sodium**	**142 mEq/L**
**Potassium**	**3.9 mEq/L**
**Chloride**	**103 mEq/L**
**Carbon Dioxide (CO**_**2**_ **)**	**28 mEq/L**
**Blood Urea Nitrogen (BUN)**	**14 mg/dL**
**Creatinine (Cr)**	**1.1 mg/dL**
**Glucose**	**105 mg/dL**

**Table t10-jetem-5-3-o1:** 

**Appearance**	**Cloudy**
**Color**	**Yellow**
**Glucose**	**Negative**
**Ketones**	**Trace**
**Sp Gravity**	**1.015**
**Blood**	**Trace**
**pH**	**6.5**
**Protein**	**4+**
**Nitrite**	**Negative**
**Leukocyte**	**Negative**
**WBC**	**0–2/high powered field (hpf)**
**Red blood cells (RBC)**	**10–15/hpf**
**Squamous Cells**	**10/hpf**
**Bacteria**	**0–2/hpf**

**Figure f1-jetem-5-3-o1:**
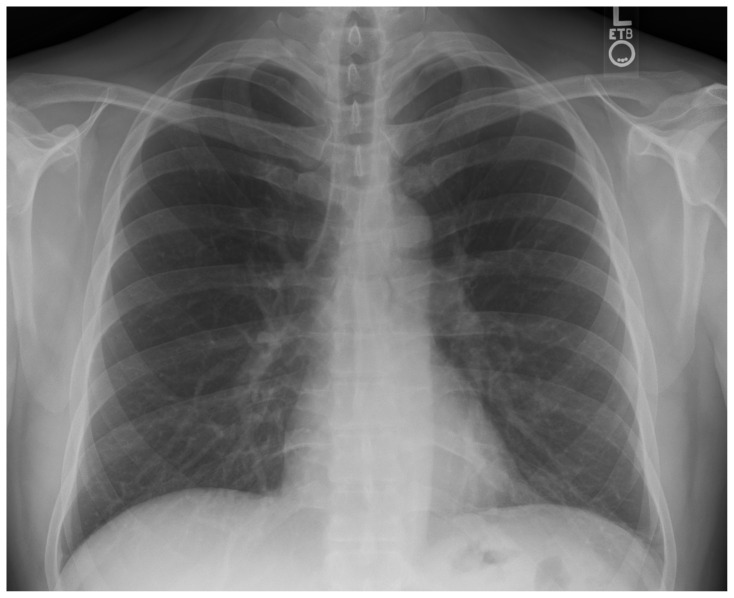


**Figure f2-jetem-5-3-o1:**
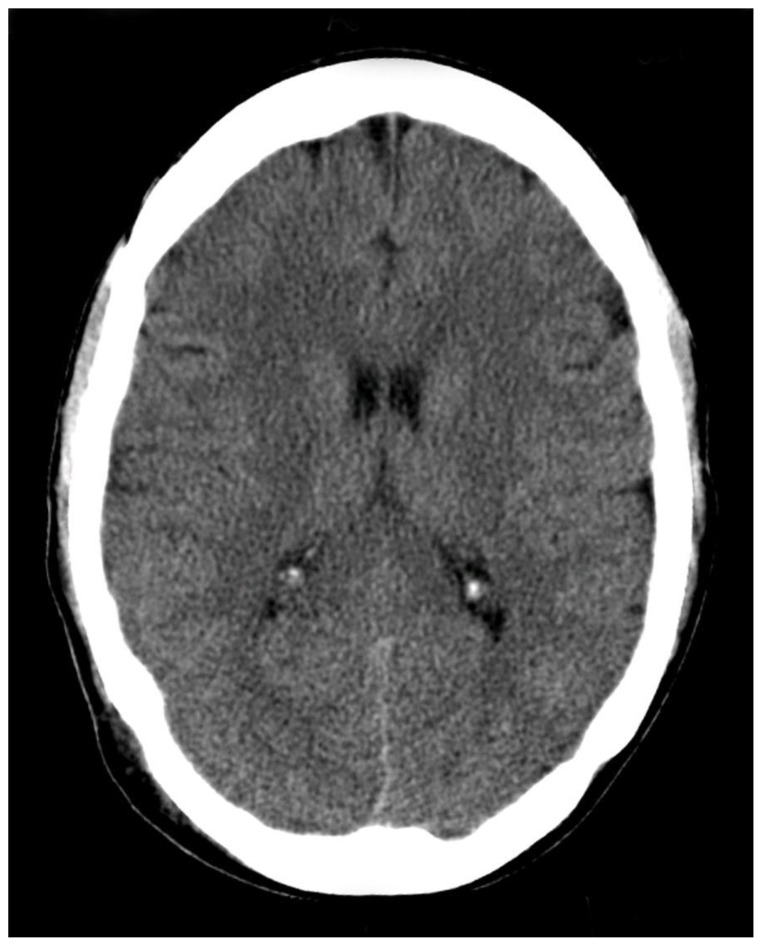


**Figure f3-jetem-5-3-o1:**
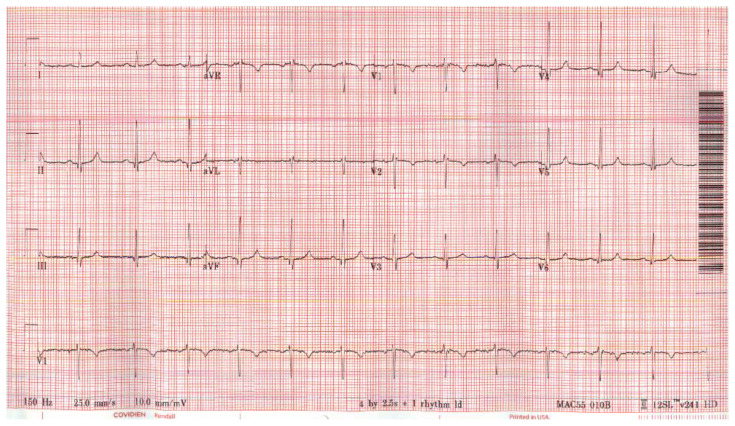


**Table t11-jetem-5-3-o1:** 

**Urine Drug Screen**	
**Amphetamines:**	**Negative**
**Barbiturates:**	**Negative**
**Benzodiazepines:**	**Negative**
**Cocaine:**	**Negative**
**Opiates:**	**Negative**
**Cannabinoids:**	**Negative**

**Table t12-jetem-5-3-o1:** 

**Serum Toxicology**	
**Acetaminophen:**	**<10 mcg/mL**
**Salicylates:**	**<15 mg/dL**
**Ethanol:**	**<10 mg/dL**

**Table t13-jetem-5-3-o1:** 

**Prothrombin Time (PT)**	**29.1 seconds**
**Partial Thromboplastic Time (PTT)**	**15.1 seconds**
**International Normalized Time (INR)**	**1.1**

**Table t14-jetem-5-3-o1:** 

**ALT**	**50 IU/L**
**AST**	**22 IU/L**
**AlkPhos**	**88 IU/L**
**T-bili**	**1.0 mg/dL**
**Direct Bilirubin**	**0.5 mg/dL**
**Mag**	**1.8 mEq/L**

## FOR EXAMINER ONLY

### Oral Case Summary

**Diagnosis: Eclampsia**

**Case Summary:** This is a 29-year-old female patient with no significant past medical history, who is presenting to the ED after suffering a seizure. The patient is somnolent and minimally responsive to verbal commands. She is unable to answer questions but is maintaining her airway. Her husband is present and states that this has never happened before. She and her husband were watching television when she became unresponsive and began to have shaking movements of her upper and lower extremities. After several minutes, she stopped shaking, and he brought her to the ED. She has recently been complaining of a headache, but it usually resolves with oral acetaminophen. She gave birth to a healthy baby boy three weeks ago, with no complications, and has otherwise been doing well while caring for him at home.

**Order of Case:** This is the case of a post-partum female patient who is presenting with eclampsia. This patient notably has had a seizure and has elevated blood pressure and severe proteinuria. If this presentation is not recognized and a magnesium-bolus/drip initiated, the patient will continue to have seizures in the ED. Benzodiazepines and other sedatives will only temporarily correct this problem. If the condition is unrecognized for ≥ 3 seizures in the ED, the patient should develop cardiac arrest. After one-round of appropriate advanced cardiac life support (ACLS) resuscitation, return of spontaneous circulation (ROSC) should be obtained. If, again, eclampsia is not recognized and treated with IV magnesium, the patient should have another cardiac arrest. ACLS should again be utilized by the learner, and ROSC obtained after one round of compressions and epinephrine administration. If eclampsia continues to go unrecognized, the examiner should suggest alternate methods to care for her seizures, such as intubation and administration of propofol or phenobarbital to induce general anesthesia, and the case should be terminated once the patient is successfully sedated and transferred to the ICU. If recognized early, the patient should stabilize and ultimately be admitted to the ICU. The examinee should be able to state that reflexes and respiratory effort are being monitored during magnesium therapy.

**Disposition: Admit to intensive care unit**

**Critical Actions:**

1. Administer 4–6 gm magnesium bolus
2. Control hypertension with parenteral hydralazine or labetalol
3. Administer magnesium-infusion, 2–4 gm/hr
4. Monitor magnesium therapy with reflex and respiratory checks
5. Admit to intensive care unit

**Dangerous Actions:**

1. Sending the patient to CT-scan after cardiac arrest without a secure airway.
2. Allowing the patient to be admitted to a medical floor.

### Historical Information

**Chief Complaint:** “She had a seizure.”

**History of present illness:** A 29-year-old female patient is brought to the ED by her husband after she became unresponsive and subsequently had shaking movements of her upper and lower extremities. The patient is unable to provide any history, and the information is obtained from her husband. He states that though she has recently been well, she intermittently has complained of headaches, and has used oral acetaminophen for relief. She has been caring for her three-week-old son, who was recently delivered vaginally, without complication.

**Past Medical history:** None

**Past Surgical history:** None

**Patient’s Medications:** Prenatal vitamins

**Allergies:** No known drug allergies

**Social history:**

- Tobacco: None
- Tobacco: Occasional
- Drug use: None

**Family history:** None

### Physical Exam Information

[Table t2-jetem-5-3-o1]**Weight:** 79 kg

**General appearance:** Somnolent, minimally rousable to noxious stimuli

**Primary survey:**

- **Airway:** Intact, no stridor, sonorous, no obvious obstruction
- **Breathing:** Lungs clear bilaterally
- **Circulation:** Intact peripheral pulses, normal heart tones, vitals as above

**Physical examination:**

- **General appearance:** Somnolent, moves extremities in response to pain
- **Head, eyes, ears, nose and throat (HEENT):**
  ○ **Head:** WNL (within normal limits)
  ○ **Eyes:** Pupils equal, 3mm bilaterally, reactive to light, no nystagmus
  ○ **Ears:** WNL
  ○ **Nose:** WNL
  ○ **Oropharynx/Throat:** Mucous membranes moist, no tongue lesions
- **Neck:** WNL
- **Chest:** Lungs clear to auscultation bilaterally, no wheezes, crackles or rales
- **Cardiovascular:** Tachycardic, but no extraneous heart sounds, normal S1/S2
- **Abdominal/GI:** Soft, non-tender, non-distended, no guarding
- **Genitourinary:** Normal external genitalia, no bleeding noted, cervical os is closed, no signs of tearing or infection
- **Rectal:** WNL
- **Extremities:** Appears to move all extremities equally, 2+ pitting edema of the bilateral lower extremities, equal pulses throughout
- **Back:** WNL
- **Neuro:** EOMI (Extraocular Movements Intact), PERRL (Pupils Equal, Round Reactive to Light), no facial asymmetry, unable to test speech, gag intact, tongue in the midline, uvula in the midline, moving extremities in response to pain, unable to adequately test strength, no obvious focal neurologic deficits noted
- **Skin:** Warm, dry, intact, no pallor
- **Lymph:** WNL
- **Psych:** Unable to test

### Critical Actions and Cueing Guidelines

1. **Administer 4–6 gm magnesium bolus.**
  - Cueing Guideline (if applicable):
    If magnesium is not bolused, the patient’s husband can ask, “How can we prevent her from seizing again?”
2. **Control hypertension with parenteral hydralazine or labetalol.**
  - Cueing Guideline (if applicable):
    If oral medications are provided, the patient may vomit the medication and require something else. If no medication provided, the RN can state, “The admission team is worried about the patient’s blood pressure; is there something we can do before transporting the patient?”
3. **Administer magnesium infusion, 2–4 gm/hour.**
  - Cueing Guideline (if applicable):
    If only a bolus is provided, the RN can ask, “Is there a way to make sure the patient’s magnesium level is high enough to prevent seizures?”
4. **Monitor magnesium therapy by checking respiratory rates and reflexes.**
  - Cueing Guideline (if applicable):
    The RN should state, “I’m training a new graduate nurse, and they are wondering how you know if the magnesium level is elevated. Are you able to explain this?”
5. **Admit to intensive care unit.**
  - Cueing Guideline (if applicable):
    If attempting to admit to an alternate location, the admitting physician should ask how to treat this condition, or if any other teams have evaluated this condition previously.

## ORAL BOARDS ASSESSMENT

*Tricyclic Antidepressant Overdose*

Learner: _________________________________________

**Critical Actions:**

□ Administer 4–6 gm magnesium bolus
□ Control hypertension with parenteral hydralazine or labetalol
□ Administer magnesium-infusion, 2–4 gm/hr
□ Monitor magnesium therapy with reflex and respiratory checks
□ Admit to intensive care unit

**Summative and formative comments:**

**Milestone assessment:** [Table t3-jetem-5-3-o1]

### Stimulus Inventory

[Table t4-jetem-5-3-o1]

#### Stimulus #1

##### Patient Information

[Table t5-jetem-5-3-o1] [Table t6-jetem-5-3-o1]

#### Stimulus #2

##### Arterial Blood Gas (ABG)

[Table t7-jetem-5-3-o1]

#### Stimulus #3

##### Complete Blood Count (CBC)

[Table t8-jetem-5-3-o1]

#### Stimulus #4

##### Basic Metabolic Panel (BMP)

[Table t9-jetem-5-3-o1]

#### Stimulus #5

##### Urinalysis

[Table t10-jetem-5-3-o1]

#### Stimulus #6

##### Chest Radiograph

[Fig f1-jetem-5-3-o1]Image source: Stillwaterising. Normal PA chest radiograph. In: Wikimedia Commons. https://commons.wikimedia.org/wiki/File:Chest_Xray_PA_3-8-2010.png. Published March, 2008. Accessed March 10, 2020. Public domain.

#### Stimulus #7

##### Non-contrast Head CT

[Fig f2-jetem-5-3-o1]Image source: Filler AG. Normal head-CT. In: Wikipedia.org https://en.wikipedia.org/wiki/File:Brain_CT_scan.jpg. Published July 2009. Accessed March 10, 2020. CC BY-SA 3.0.

#### Stimulus #8

##### Electrocardiogram

[Fig f3-jetem-5-3-o1]Author’s own image

#### Stimulus #9

##### Toxicology

[Table t11-jetem-5-3-o1] [Table t12-jetem-5-3-o1]

#### Stimulus #10

##### Coagulation Panel

[Table t13-jetem-5-3-o1]

#### Stimulus #11

##### Liver function panel

[Table t14-jetem-5-3-o1]

## DEBRIEFING AND EVALUATION PEARLS

### Eclampsia

1. Pre-eclampsia is thought to be a placental disorder of implantation. This leads to reduced blood flow and placental ischemia resulting in endothelial dysfunction and eventually pre-eclampsia, intrauterine growth restriction and preterm birth.^2^
2. Pre-eclampsia is defined as new onset hypertension measured as systolic >140 mmHg and diastolic >90 mmHg accompanied by one or more other features: proteinuria, maternal dysfunction of liver, kidney, central nervous system or hematological involvement. Though pre-eclampsia and eclampsia typically manifest during pregnancy after 20 weeks estimated gestational age, the disease can be seen as late as 6 weeks postpartum. 5, 6
3. Differential diagnosis should also include chronic hypertension and gestational hypertension. When seizures are seen, consider epilepsy, hypoglycemia, or medications that induce seizures or lower seizure threshold.^6^
4. Maternal organ dysfunction as a result of pre-eclampsia can also include uteroplacental dysfunction that may present as fetal growth restriction or abnormal Doppler ultrasound findings of uteroplacental blood flow. Eclampsia is the progression of this disease process that manifests as maternal seizures. 6, 7
5. Goals in treatment: aggressive treatment of hypertensive emergency with antihypertensive therapy, maternal seizure prevention in severe pre-eclampsia with magnesium sulfate, and limiting injury to the fetus. If treatment fails to correct the maternal hypertension, the only treatment of the disease process is delivery. 6
6. Antihypertensive therapy includes labetalol (10–20 mg IV, q 20–30 minutes) and hydralazine (5–10 mg IV, q 20 minutes) bolus dosing as first line therapy. Oral nifedipine (30 mg PO) can be given if intravenous access is not available. The goal is to lower maternal blood pressure 15%–20%, with a goal SBP of 140–150 mmHg and DBP of 90–100 mmHg.^6^, ^8^
7. Magnesium sulphate is first line therapy for treatment and prophylaxis of maternal seizures with the helpful side effect of lowering blood pressure. Given as a 6 g loading dose followed by a 2 g/hour infusion, patients require close monitoring for respiratory and cardiac failure.^6^
